# Comparison of Inter-Method Agreement and Reliability for Automatic Brain Volumetry Using Three Different Clinically Available Software Packages

**DOI:** 10.3390/medicina60050727

**Published:** 2024-04-27

**Authors:** Kwang Ho Choi, Young Jin Heo, Hye Jin Baek, Jun-Ho Kim, Jeong Yoon Jang

**Affiliations:** 1 Department of Thoracic and Cardiovascular Surgery, Research Institute for Convergence of Biomedical Science and Technology, Pusan National University Yangsan Hospital, Pusan National University School of Medicine, 20 Geumo-ro, Mulgeum-eup, Yangsan-si 50612, Republic of Korea; 2 Department of Radiology, Busan Paik Hospital, Inje University College of Medicine, 75, Bokji-ro, Busanjin-gu, Busan 47392, Republic of Korea; 3 Department of Radiology, Gyeongsang National University School of Medicine and Gyeongsang National University Changwon Hospital, 11 Samjeongja-ro, Seongsan-gu, Changwon 51472, Republic of Korea; 4 Miracle Radiology Clinic, 201 Songpa-daero, Songpa-gu, Seoul 05854, Republic of Korea; 5 Department of Electrical and Electronic Engineering, Yonsei University, Seoul 03722, Republic of Korea; 6 Division of Cardiology, Department of Internal Medicine, Gyeongsang National University School of Medicine and Gyeongsang National University Changwon Hospital, 11 Samjeongja-ro, Seongsan-gu, Changwon 51472, Republic of Korea

**Keywords:** brain volumetry, NeuroQuant, Freesurfer, Heuron AD, reliability, memory deficits

## Abstract

*Background and Objectives:* No comparative study has evaluated the inter-method agreement and reliability between Heuron AD and other clinically available brain volumetric software packages. Hence, we aimed to investigate the inter-method agreement and reliability of three clinically available brain volumetric software packages: FreeSurfer (FS), NeuroQuant^®^ (NQ), and Heuron AD (HAD). *Materials and Methods:* In this study, we retrospectively included 78 patients who underwent conventional three-dimensional (3D) T1-weighed imaging (T1WI) to evaluate their memory impairment, including 21 with normal objective cognitive function, 24 with mild cognitive impairment, and 33 with Alzheimer’s disease (AD). All 3D T1WI scans were analyzed using three different volumetric software packages. Repeated-measures analysis of variance, intraclass correlation coefficient, effect size measurements, and Bland–Altman analysis were used to evaluate the inter-method agreement and reliability. *Results:* The measured volumes demonstrated substantial to almost perfect agreement for most brain regions bilaterally, except for the bilateral globi pallidi. However, the volumes measured using the three software packages showed significant mean differences for most brain regions, with consistent systematic biases and wide limits of agreement in the Bland–Altman analyses. The pallidum showed the largest effect size in the comparisons between NQ and FS (5.20–6.93) and between NQ and HAD (2.01–6.17), while the cortical gray matter showed the largest effect size in the comparisons between FS and HAD (0.79–1.91). These differences and variations between the software packages were also observed in the subset analyses of 45 patients without AD and 33 patients with AD. *Conclusions:* Despite their favorable reliability, the software-based brain volume measurements showed significant differences and systematic biases in most regions. Thus, these volumetric measurements should be interpreted based on the type of volumetric software used, particularly for smaller structures. Moreover, users should consider the replaceability-related limitations when using these packages in real-world practice.

## 1. Introduction

Magnetic resonance imaging (MRI)-based brain volumetry is increasingly being applied for assessing a wide range of neurological diseases, specifically neurodegenerative diseases such as Alzheimer’s disease (AD), in clinical practice [1]. In contrast to the visual assessment of brain volume changes, the quantitative assessment of brain volume serves as a valid biomarker of the clinical state and disease progression by providing reliable and robust inferences regarding the underlying disease-related mechanisms [1,2].

Various volumetric software packages, regardless of their commercialization status, have been developed and used for clinical and research purposes. These software packages automatically measure regional brain volumes or cortical thicknesses in a simpler and more intuitive manner over time [2,3]. With the increasing use of volumetric software, several studies have compared NeuroQuant^®^ (NQ) and FreeSurfer (FS) as representative products for brain volumetry [4,5,6,7]. NQ has several advantages over FS, such as shorter total processing time, user-friendly workflow, and direct interaction with the PACS server. For example, the server can be set up to interface directly with the MRI scanner, allowing automatic sending of information for processing. In addition, NQ has an integrated normal control database that is better suited for routine clinical applications [8]. On the other hand, FS is freely available, adaptable, and commonly used in research settings as users can manually edit any errors detected in the automated region of interest (ROI) and perform additional volumetric analyses through further manual segmentation of the ROI [9]. In previous studies, significant differences have been observed in certain volume measurements between the two methods, as NQ provided larger volumes of brain regions than FS for large structures such as the intracranial volume, forebrain parenchyma, lateral ventricles, and cerebellum [4,5,6,7,9]. In real-world practice, automated brain volumetry improves diagnostic accuracy across various fields, such as clinical psychiatry and neurology, where diagnosis often relies on subjective self-reports and test results [10,11]. However, several limitations are associated with potential errors that occur during the quantitative analyses of brain volumes. These limitations can stem from physical constrains, the lack of large-scale normal data, and pathophysiological constrains [12]. Reproducibility poses another challenge in the clinical application of different volumetric software packages for interpreting the measured brain volumes. Although the results of recent studies revealed good-to-excellent correlations between different volumetric software packages, significant differences were observed between the measured brain volumes. This underscores the need for careful attention during interpretation [3,7,12,13,14].

In the past few decades, beginning in early 2000s, various brain volumetry software packages with unique characteristics have been rapidly developed by multiple international vendors to compensate for the shortcomings of existing software products. Heuron AD (HAD) is a recently approved deep learning-based volumetric software package developed by the Ministry of Food and Drug Safety (MFDS, or Korea Food and Drug Administration [K-FDA]). HAD employs a segmentation model that uses deep neural networks. This software package provides information on neurodegeneration by comparing age-adjusted volume and cortical thickness measurements with clinically normative data, and indicates the presence and location of brain atrophy. Additionally, HAD includes a longitudinal analysis function that allows the analysis of repeated MRI scans to measure and monitor the changes in cortical thickness over time. At our institution, HAD was used for brain volume analysis, which was provided to us for a limited time as a product demo. This prompted us to compare the brain volume analysis results of the same patients obtained from FS and NQ with those from HAD. To date, no comparative study has evaluated the inter-method agreement and reliability between HAD and other clinically available software packages. We hypothesized that any differences or systemic bias in volume measurements in certain brain structures between the different software packages could warrant caution regarding the reliability of automated brain segment analysis and the interpretation of the results. Thus, in this study, we aimed to evaluate the inter-method agreement and reliability of three clinically available software packages: the established research software, FS, the commonly used commercial software, NQ, and the recently developed commercial software, HAD.

## 2. Material and Methods

### 2.1. Patients

This retrospective study involving human participants was conducted in accordance with the ethical standards of the Institutional Research Committee and the 1964 Declaration of Helsinki and its later amendments or comparable ethical standards. This study was approved by the Institutional Review Board of Gyeongsang National University Changwon Hospital (Approved Protocol Code: GNUCH 2022-11-026; Approval Date: 14 December 2022). The requirement for informed consent was waived owing to the retrospective nature of the study. Patient records and information were anonymized and de-identified before data analysis.

We searched the picture archiving and communication systems and electronic medical records of patients who underwent brain MRI, including conventional 3D T1-weighted imaging (T1WI), for the assessment of memory impairment. The characteristics of the study population are listed in Table 1. A total of 78 patients (52 women and 26 men; age range: 21–88 years; mean age: 66.2 ± 17.4 years) were included in this study. Among the 78 patients with subjective cognitive impairment (i.e., memory impairment), 45 were categorized into the non-AD group (normal objective cognitive function [21/45, 46.7%] or mild cognitive impairment [MCI; 24/45, 53.3%]), while the remaining 33 were categorized into the AD group. The diagnoses of MCI and AD were clinically determined by three dementia specialists (two neurologists and one psychiatrist) using the following neuropsychiatric evaluations tools: Mini-Mental State Examination, Clinical Dementia Rating scale, Seoul Neuropsychological Screening Battery, Consortium to Establish a Registry for Alzheimer’s Disease, Diagnostic and Statistical Manual of Mental Disorders (5th edition) criteria [15], and the National Institute of Neurological and Communicative Disorders and Stroke and the Alzheimer’s Disease and Related Disorders Association criteria [16].

### 2.2. Image Acquisition

MRI was performed using a 3T system (Signa^TM^ Architect; GE Healthcare, Milwaukee, WI, USA) with a 48-channel head coil. In addition to 3D T1WI, routine brain MRI (axial T2-weighted imaging, fluid-attenuated inversion recovery, diffusion-weighted imaging, and 3D multi-echo gradient echo [susceptibility-weighted angiography]) was performed in all patients. Meanwhile, 3D T1WI (BRAVO) scans were obtained with sagittal planes covering the whole brain and using the following parameters: repetition time/echo time, 7.0/2.8; section thickness, 1.0 mm; matrix, 210 × 210 mm; flip angle, 12.0; field of view, 210 × 210 mm; parallel imaging acceleration factor, phase 2; bandwidth, 31.25; and acquisition time, 3 min 4 s.

### 2.3. Image Post-Processing Volumetric Procedures

Following the visual inspection of the scans by a faculty neuroradiologist (H.J.B., with 13 years of post-training experience) to identify the presence of artifacts that could affect post-processing, the raw Digital Imaging and Communications in Medicine (DICOM) data were submitted and analyzed using three different processing pipelines: NQ (CorTechs Labs, San Diego, CA, USA), FS (Harvard University, Boston, MA, USA), and HAD (Heuron Co., Ltd., Seoul, Republic of Korea). Two faculty neuroradiologists (H. J. B., with 13 years of post-training experience and Y.J.H., with 8 years of post-training experience) performed the automated analyses using NQ and HAD in all patients, and a software engineer jointly executed the automated analyses using FS in all patients. The software packages used in this study provided the total intracranial volume and the volumes of the cortical gray matter (GM), cerebral white matter (WM), hippocampus, amygdala, caudate nucleus, putamen, pallidum, thalamus, and cerebellum.

NQ is the first FDA-approved volumetry software package and a standalone, fully automatic processing pipeline. In NQ, the brain is inflated to a spherical shape, and mapped to a common spherical space using the Talairach atlas coordinates. The segmented brain regions are then identified, and the brain is deflated to its original shape. The volume of each brain region is corrected for head size differences by normalizing it to the intracranial volume (ICV), and the resulting output is expressed as a percentage. The results are compared with the data from healthy controls, which have been stored in the NQ database.

FS uses a template-driven approach for volumetric and surface-based segmentation, as described in previous studies [9,16,17,18]. All data were batch-processed using an Intel i7-10700 central processing unit (CPU) running VirtualBox (centos7), and the data were initially processed using the *recon-all* command to produce fully segmentation.

HAD provides information about brain atrophy. The software divides the brain region, calculates the volume and cortical thickness, and compares the calculated results with normative data to provide a brain atrophy index for users. The segmentation engine introduced a deep learning architecture to segment the entire brain into 98 ROIs using a state-of-the-art (SOTA) parcellation model consisting of three fully convolutional neural networks (FCNNs) and an aggregation layer. Each FCNN computes the geometrical features in the axial, coronal, and sagittal slices. The features from the three FCNNs are aggregated by the final layer to create a parcellation mask. All the training data for the parcellation model were manually annotated by expert neurologists.

### 2.4. Statistical Analysis

Normality of data was assessed using the Kolmogorov–Smirnov test, and data were expressed as the mean ± standard deviation. Repeated-measures analysis of variance (ANOVA), followed by post-hoc tests with Bonferroni correction for multiple comparisons, were performed to assess differences in the mean volume measurements between NQ, FS, and HAD. The inter-method agreement across the three software packages was assessed using the intraclass correlation coefficient (ICC) values, which were interpreted as follows: 0.01–0.20, slight; 0.21–0.40, fair; 0.41–0.60, moderate; 0.61–0.80, substantial; and 0.81–1.00, almost perfect [18]. The effect sizes were used to evaluate the inter-software agreements across the three software packages in measuring the volume using the following equation: effect size = mean difference/pooled standard deviation [5,19]. The effect sizes were categorized as follows: <0.20, negligible; 0.2–0.49, small; 0.50–0.79, medium; and >0.8, large [20]. Bland–Altman plots were generated, and the mean bias and 95% limits of agreement (LOA) were obtained for each comparison. All statistical analyses were performed using statistical software packages (SPSS, version 26.0, IBM, Armonk, NY, USA; MedCalc, version 19.8, MedCalc Software, Mariakerke, Belgium), and a *p* value of <0.05 (two-sided) was considered to indicate significance.

## 3. Results

### 3.1. Comparison of Total ICV

A significant difference was observed in the total ICV between the three software packages (Table 2). The total ICV obtained using NQ was the largest among the measures obtained from all patients (NQ: 1427.37 ± 152.94 cm^2^, FS: 1414.41 ± 142 cm^2^, HAD: 1381.46 ± 140.69 cm^2^) (*p* < 0.0001). However, no significant difference was observed between NQ and FS in all patients (*p* = 0.101). All total ICVs showed almost perfect agreement (0.926–0.967), with a negligible-to-small effect size between the three software packages. In the Bland–Altman analysis, the mean bias and 95% LOA between NQ and HAD were greatest across all the software comparisons (45.90 cm^3^ [–104.97 cm^3^ and 196.78 cm^3^] (Supplementary Material Table S1).

### 3.2. Comparison of the Measured Volumes of Segmented Brain Regions

NQ showed the largest measured volume in the cortical gray matter (GM), cerebral white matter (WM), putamen, thalamus, and cerebellum. However, NQ showed the smallest measured volume in the pallidus. HAD showed the largest measured volume in the hippocampus, amygdala, and caudate.

**Figure 1:**
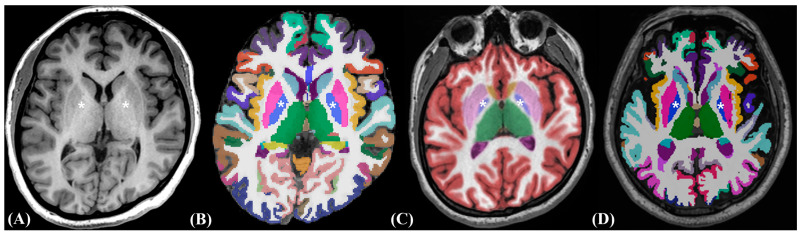
Representative color-coded axial MR images at the level of the basal ganglia. An axial T1-weighted image (**A**) is shown at the basal ganglia level with color-coded images of the FS, NQ, and HAD. In these representative images, the pallidum appears smaller in NQ (**C**) than in FS (**B**) or HAD (**D**). The pallidum is indicated with asterisks. FS, FreeSurfer; HAD, Heuron AD; NQ, NeuroQuant.

According to the repeated-measures ANOVA, the three software packages showed significant differences in the measured volume for most brain regions (Table 3). The measured volumes of most brain regions were significantly different between NQ and FS, and two software packages showed almost perfect agreement in most regions (Table 4). With regard to the effect size, the pallidum showed the largest effect size in both hemispheres (Table 5, and Figure 1).

The comparison between NQ and HAD also showed significant differences in volumetric measurements for most regions, except for the amygdala (Lt., *p* = 0.634). NQ and HAD showed substantial to almost perfect agreement for all individual regions, except for both pallidi (ICC: 0.37–0.56), and the pallidum also showed the largest effect size in both hemispheres (Figure 1C,D).

Comparison between FS and HAD showed no significant differences in the volumetric measurements of the deep GM. FS and HAD revealed substantial to almost perfect agreement for all individual regions, in contrast to the results between NQ and HAD. The largest effect size was observed in cortical GM (Lt. = 0.97; Rt. = 0.93).

The results of the Bland–Altman analysis of all software comparisons are summarized in the Supplementary Material Table S2. The mean bias and 95% LOA of the cortical GM were the greatest among the segmented brain regions in the comparison between NQ and HAD and between FS and HAD. However, the mean bias and 95% LOA for cerebral WM were the greatest in the comparison between NQ and FS.

### 3.3. Results of Subgroup Analyses by Presence or Absence of AD

According to the subgroups (non-AD vs. AD), the total ICV also showed significant differences between the three software packages (Table 2). The total ICV obtained using the NQ was the largest in non-AD patients (NQ: 1473.69 ± 158.79 cm^2^, FS: 1446.49 ± 144.85 cm^2^, and HAD: 1420.02 ± 139.01 cm^2^; all *p* < 0.0001). By contrast, the total ICV obtained using FS was the largest in AD patients (FS: 1370.66 ± 127.97 cm^2^, NQ: 1364.21 ± 120.35 cm^2^, HAD: 1328.89 ± 126.94 cm^2^; *p* < 0.0001). However, no significant difference was found between the NQ and FS in AD patients (*p* = 1.000). In addition, the total ICV showed almost perfect agreement (0.894–0.992), with a negligible-to-small effect size, regardless of the disease status or type of software compared. For mean bias with 95% LOA, the values between NQ and HAD were the greatest among all the software comparisons: 53.67 cm^3^ [127.12 cm^3^ to 234.46 cm^3^] in non-AD patients and 35.32 cm^3^ [−59.88 to 130.53] in AD patients (Supplementary Material Table S1).

The comparison between NQ and HAD also showed significant differences in the volumetric measurements of most regions, except the amygdala (Lt.: *p* = 1.000 in non-AD patients vs. *p* = 0.136 in AD patients; and Rt., 1.000 in AD patients) and right caudate nucleus (*p* = 1.000 in AD patients). NQ and HAD showed substantial to almost perfect agreement for all individual regions, except for both pallidi (ICC, 0.38–0.61) and hippocampi (ICC, Lt. = 0.600, Rt. = 0.523 in non-AD patients). Regardless of the AD, the pallidum showed the largest effect size in both hemispheres (*d* = 2.01–6.17).

The comparison between FS and HAD showed no significant differences in volumetric measurements of the deep GM (the left pallidum and both thalami in non-AD patients; and the right hippocampus, left caudate, left pallidum, both putamina, and both thalami in patients with AD). FS and HAD revealed substantial to almost perfect agreement for all individual regions, except for both pallidi (ICC, 0.23–0.92). The largest effect size was observed in the cortical GM of AD patients with AD (Lt., 1.91; Rt., 1.83) and in the right putamen (1.61) of patients without AD.

Supplementary Material Table S2 summarizes the results of the Bland–Altman analysis for all software comparisons. The mean bias and 95% LOA for the cortical GM were the greatest among the segmented brain regions in the comparison between NQ and HAD and between FS and HAD in both subgroups. However, the mean bias and 95% LOA for the cerebral WM were the greatest in the comparison between NQ and FS, except in the left hemisphere in the non-AD group.

## 4. Discussion

In this study, we compared the inter-method agreement and reliability between three clinically available brain volumetry software packages: FS, NQ, and HAD. We found substantial to almost perfect agreement for most segmented brain regions, except for the pallidi. However, the volume measurements for most segmented regions showed significant differences and moderate or large effect sizes across the three volumetric software packages. In particular, both pallidi showed the largest effect size in the comparison between NQ and FS and between NQ and HAD. Meanwhile, the cortical GM showed the largest effect size in the comparison between FS and HAD. In the current study, the favorable inter-method agreement for most segmented brain regions suggested that each software package provided good qualitative information on the brain structure. However, the significant differences and systemic biases in the majority of brain volume measurements, likely stemming from procedural variations in each method, can raise doubts on the reliability of automated brain analysis as a quantitative tool for routine clinical practice.

Various volumetric software packages have recently become clinically available for automatic segmentation in clinical settings. Several previous studies have extensively explored the inter-method reliability for various volumetric software, especially those comparing FS and NQ [5,6,7,12,13,21]. These studies reported good-to-excellent correlations in the volumetric results across various brain regions. However, they also observed significant overall differences in the mean volumes for segmented brain regions, which was consistent with the results of the present study [5,6,7,21]. Interestingly, they observed larger volumes when using NQ compared with FS in most brain regions, which was similar to the results of this study, except for both hippocampi, left amygdala, and pallidi. However, to the best of our knowledge, no clinical studies have explored HAD, as it was developed more recently. Although NQ provided the largest volume measurements for most brain regions, HAD showed the largest volume measurements in the hippocampus, amygdala, and caudate.

Significant mean differences, as shown in our study, have also been previously reported in the volume measurements of most brain regions obtained using different software packages [12,22]. Although Inbrain^®^ (MIDAS Information Technology Co., Ltd., Seongnam, Republic of Korea) and NQ showed good-to-excellent inter-method reliability for all brain regions, they also showed significantly different volume measurements with large effect sizes [13]. Another study [23] also demonstrated good-to-excellent inter-method reliability and correlation between vendor-provided volumetry software and NQ for most brain structures. However, a significant difference was found in the measured volumes, except for the right hippocampus. This variation may be attributed to the differences in volumetric results obtained using various software packages based on different atlases [23,24]. Furthermore, image noise and heterogeneity in intensity could introduce errors in quantitative measurements and affect the performance of the volumetry software [22].

In this study, NQ showed significantly smaller pallidus volume than FS and HAD. Consistent with our findings, previous studies have also shown a stronger correlation between the measurements of large structures (such as the ICV) and a lower correlation between the measurements of small and deeper structures in NQ compared with other available volumetric software packages [5,6,12,23]. Among these small structures, the pallidum showed the lowest correlation across the volumetric software packages and the largest effect size among the segmented regions. Hence, previous studies have proposed two main explanations for the inconsistency in pallidal volume measurements. First, the accurate segmentation of the pallidum from the adjacent WM is challenging owing to its T1 signal intensity, which is influenced by the higher myelination content of the pallidum. This challenge could be addressed by including the adjacent WM and putamen in the calculation [5,12,25]. Second, metal deposition associated with the aging or degeneration processes in the pallidum may affect the T1 relaxation time, thereby impacting software-based volume measurement [26]. However, another previous study demonstrated good reproducibility of pallidum measurements between FS and Inbrain^®^ owing to their similar segmentation method [27]. Furthermore, our analysis revealed that the measured volumes of the pallidum, putamen, and thalamus with HAD were closer to the FS values than the NQ values, regardless of statistical significance. In addition, no significant differences and a higher ICC were observed in the measurements of both pallidi between FS and HAD than between NQ and HAD or between NQ and FS in the present study.

The volume of the hippocampus is considered an important biomarker of AD [28] and has predictive value for the conversion of MCI to AD in clinical practice [7]. Although the prognostic value of the hippocampal measurements was not investigated in this study, we measured and compared the volume of this structure obtained using the three software packages and also conducted group comparisons of these values in relation to the presence of AD. The largest mean hippocampal volume was obtained with HAD, followed by FS and NQ, regardless of the disease status. However, the hippocampal volumes in the AD group were consistently smaller than those in the non-AD group, regardless of the volumetric software package.

This study had several limitations. First, we retrospectively evaluated a relatively small and heterogeneous cohort of patients from a single institution, introducing a potential selection bias. Second, we classified patients with MCI into a non-AD group and dichotomized the study according to the presence of AD for group comparison owing to the small number of patients enrolled. Thus, additional studies with a larger sample size are warranted to validate our results regarding the comparison of three clinical groups using three or more volumetric software packages, as the early detection of MCA holds clinical significance as a prodromal state rather than an overt state of AD. Third, we only evaluated the inter-method reliability using a single MR scanner with a homogeneous protocol in a single institution. Therefore, multicenter studies using different scanning environments and protocols are required to confirm our results. Fourth, age could potentially influence the volumes in various brain regions; however, we were unable to obtain age-adjusted values using all software packages. Finally, although FS has shown accuracy and reliability comparable to those of manual segmentation performed by experts in previous studies, the lack of a reference standard for true brain volumes of the various anatomic regions remains a limitation [29,30,31].

## 5. Conclusions

In conclusion, we compared the measured volumes of various brain regions using three clinically available volumetric software packages, FS, NQ, and HAD. We observed substantial to almost perfect agreement between the software packages. However, significant differences were observed in the mean volumes for most brain regions and consistent systematic biases with wide LOA between the three software packages in both AD and non-AD groups, potentially limiting reproducibility. Unfortunately, no objective gold standard has been established for measuring brain segment volume, making it challenging to determine which software is closest to reality. Previous studies have used various methods and tools to measure brain volumes, further complicating comparisons. Similar to previous studies, our results are unsuitable for determining which software package is superior for evaluating patient conditions for clinical and research purposes. However, our findings underscore the importance of interpreting the volumetric measurements obtained using different software packages cautiously in real-world practice. All users, including clinicians and researchers, should be aware of these inherent limitations related to the replaceability of various volumetric software packages when using them in clinical settings, e.g., in tracking changes for longitudinal analyses.

## Figures and Tables

**Table 1:** Demographic data of the study participants.

	Normal (*n* = 21)	MCI (*n* = 24)	AD (*n* = 33)
Age, years ^a^	48.7 ± 18.1 (range: 21–78)	75.7 ± 6.93 (range: 63–85)	74.5 ± 7.81 (range: 58–89)
Sex			
Female	10 (47.6%)	15 (62.5%)	27 (81.8%)
Male	11 (52.4%)	9 (37.5%)	6 (18.2%)
MMSE score ^a^	29.56 ± 1.42	27.83 ± 1.61	20.19 ± 4.36
CDR ^a^	0.01 ± 0.07	0.52 ± 0.27	0.86 ± 0.41

Note—^a^ Data are presented as mean ± standard deviation. Otherwise, data represent the number of patients. AD, Alzheimer’s disease; CDR, clinical dementia rating; MCI, mild cognitive impairment; MMSE, Mini-Mental State Examination.

**Table 2:** Comparison of total intracranial volume.

	NQ	FS	HAD	*p*	NQ vs. FS			NQ vs. HAD			FS vs. HAD		
					*p*	ICC	d	*p*	ICC	d	*p*	ICC	d
Total	1427.37 ± 152.94	1414.41 ± 142.18	1381.46 ± 140.69	<0.001	0.101	0.967 (0.948–0.979)	0.088	<0.001	0.926 (0.885–0.953)	0.312	<0.001	0.961 (0.939–0.975)	0.233
Non-AD	1473.69 ± 158.79	1446.49 ± 144.85	1420.02 ± 139.01	<0.001	0.007	0.965 (0.936–0.981)	0.179	0.04	0.894 (0.808–0.942)	0.360	0.040	0.937 (0.886–0.966)	0.186
AD	1364.21 ± 120.35	1370.66 ± 127.97	1328.89 ± 126.94	<0.001	1.000	0.971 (0.941–0.986)	0.052	<0.001	0.960 (0.919–0.980)	0.286	<0.001	0.992 (0.983–0.996)	0.286

Note—Data are presented as mean ± standard deviation and intraclass correlation coefficient with 95% confidence interval and effect size (d). AD, Alzheimer’s disease; FS, FreeSurfer; HAD, Heuron AD; NQ, NeuroQuant.

**Table 3:** Comparison of measured volumes across the three volumetry software packages.

	Lt. Hemisphere						Rt. Hemisphere					
	NQ	FS	HAD	NQ vs. FS *	NQ vs. HAD *	FS vs. HAD *	NQ	FS	HAD	NQ vs. FS *	NQ vs. HAD *	FS vs. HAD *
**Cortical GM**												
Total	230.15 ± 35.40	214.27 ± 24.32	188.19 ± 29.46	<0.001	<0.001	<0.001	230.44 ± 35.13	213.13 ± 24.80	188.23 ± 28.59	<0.001	<0.001	<0.001
Non-AD	245.44 ± 35.96	223.66 ± 24.34	202.75 ± 28.21	<0.001	<0.001	<0.001	245.15 ± 36.00	223.38 ± 24.30	203.06 ± 26.61	<0.001	<0.001	<0.001
AD	209.30 ± 21.44	201.48 ± 17.77	168.32 ± 16.99	<0.001	<0.001	<0.001	210.37 ± 21.62	199.15 ± 17.86	168.00 ± 16.18	<0.001	<0.001	<0.001
**Cerebral WM**												
Total	210.82 ± 25.73	193.82 ± 25.73	203.10 ± 27.13	<0.001	<0.001	<0.001	214.86 ± 25.70	192.46 ± 25.70	205.20 ± 28.14	<0.001	<0.001	<0.001
Non-AD	216.22 ± 27.22	199.22 ± 27.22	210.94 ± 27.26	<0.001	<0.001	<0.001	220.34 ± 27.61	197.94 ± 27.61	213.26 ± 28.53	<0.001	<0.001	<0.001
AD	203.47 ± 21.84	186.46 ± 21.84	192.42 ± 23.35	<0.001	<0.001	<0.001	207.39 ± 21.03	184.99 ± 21.03	194.21 ± 23.88	<0.001	<0.001	<0.001
**Hippocampus**												
Total	3.37 ± 0.80	3.60 ± 0.59	3.93 ± 0.44	<0.001	<0.001	<0.001	3.52 ± 0.91	3.81 ± 0.65	4.08 ± 0.45	<0.001	<0.001	<0.001
Non-AD	3.73 ± 0.80	3.83 ± 0.60	4.07 ± 0.38	0.378	0.005	0.002	3.93 ± 0.86	4.05 ± 0.57	4.15 ± 0.40			
AD	2.87 ± 0.49	3.28 ± 0.41	3.75 ± 0.45	<0.001	<0.001	<0.001	2.97 ± 0.66	3.47 ± 0.61	3.99 ± 0.50	<0.001	0.021	0.259
**Amygdala**												
Total	1.48 ± 0.35	1.34 ± 0.29	1.51 ± 0.39	<0.001	0.634	<0.001	1.42 ± 0.30	1.52 ± 0.25	1.67 ± 0.34	<0.001	<0.001	<0.001
Non-AD	1.58 ± 0.39	1.44 ± 0.26	1.59 ± 0.43	<0.001	1.000	<0.001	1.53 ± 0.33	1.63± 0.21	1.83 ± 0.29	0.024	<0.001	<0.001
AD	1.34 ± 0.24	1.19 ± 0.27	1.40 ± 0.31	<.001	0.136	<0.001	1.26 ± 0.17	1.38 ± 0.21	1.46 ± 0.28	1.000	1.000	1.000
**Caudate**												
Total	3.17 ± 0.73	3.27 ± 0.51	3.41 ± 0.48	0.129	<0.001	<0.001	3.30 ± 0.71	3.23 ± 0.44	3.51 ± 0.49	0.910	<0.001	<0.001
Non-AD	2.99 ± 0.72	3.18 ± 0.52	3.26 ± 0.43	0.018	0.003	0.322	3.18 ± 0.71	3.19 ± 0.40	3.40 ± 0.46	1.000	0.008	<0.001
AD	3.42 ± 0.68	3.39 ± 0.49	3.62 ± 0.48	1.000	0.019	<0.001	3.46 ± 0.69	3.29 ± 0.48	3.66 ± 0.50	0.221	1.000	1.000
**Putamen**												
Total	5.62 ± 0.87	4.17 ± 0.72	4.00 ± 0.64	<0.001	<0.001	0.001	5.44 ± 0.91	4.21 ± 0.72	3.99 ± 0.67	<0.001	<0.001	<0.0001
Non-AD	5.89 ± 0.89	4.41 ± 0.75	4.13 ± 0.66	<0.001	<0.001	<0.001	4.47 ± 0.73	3.19 ± 0.40	4.14 ± 0.73	<0.001	<0.001	<0.0001
AD	5.25 ± 0.69	3.83 ± 0.53	3.83 ± 0.59	<0.001	<0.001	1.000	5.01 ± 0.72	3.85 ± 0.53	3.78 ± 0.53	<0.001	<0.001	0.263
**Pallidum**												
Total	0.49 ± 0.16	1.92 ± 0.32	1.88 ± 0.32	<0.001	<0.001	0.512	0.46 ± 0.14	1.88 ± 0.30	1.85 ± 0.32	<0.001	<0.001	1.000
Non-AD	0.52 ± 0.18	1.87 ± 0.32	1.89 ± 0.32	<0.001	<0.001	1.000	0.50 ± 0.16	1.83 ± 0.24	1.90 ± 0.30	<0.001	<0.001	0.010
AD	0.45 ± 0.10	2.00 ± 0.30	1.87 ± 0.31	<0.001	<0.001	0.022	0.41 ± 0.89	1.94 ± 0.36	1.78 ± 0.33	<0.001	<0.001	0.068
**Thalamus**												
Total	7.44 ± 1.12	6.69 ± 0.94	6.38 ± 0.86	<0.001	<0.001	1.000	7.25 ± 1.01	6.13 ± 0.92	6.18 ± 0.85	<0.001	<0.001	0.159
Non-AD	7.81 ± 1.21	6.70 ± 1.04	6.68 ± 0.95	<0.001	<0.001	1.000	7.65 ± 1.09	6.50 ± 0.98	6.52 ± 0.90	<0.001	<0.001	1.000
AD	6.93 ± 0.72	5.97 ± 0.57	5.98 ± 0.50	<0.001	<0.001	1.000	6.71 ± 0.54	5.63 ± 0.54	5.72 ± 0.48	<0.001	<0.001	1.000
**Cerebellum**												
Total	60.91 ± 7.41	60.18 ± 6.88	56.02 ± 7.95	0.001	<0.001	<0.001	61.89 ± 7.67	60.57 ± 7.46	57.80 ± 7.77	<0.001	<0.001	<0.001
Non-AD	63.78 ± 6.39	62.93 ± 5.91	59.42 ± 6.30	0.012	<0.001	<0.001	64.38 ± 6.97	63.20 ± 6.27	60.48 ± 6.48	0.004	<0.001	<0.001
AD	56.99 ± 6.96	56.42 ± 6.38	51.39 ± 7.70	0.105	<0.001	<0.001	58.49 ± 7.35	56.98 ± 7.54	54.14 ± 7.98	<0.001	<0.001	<0.001

Note—Data are presented as mean ± standard deviation. Bonferroni correction for multiple comparisons was applied for all *p* values of pairwise comparisons (between two software programs). * The overall *p* value calculated using repeated measures ANOVA. AD, Alzheimer’s disease; FS, FreeSurfer; GM, gray matter; HAD, Heuron AD; NQ, NeuroQuant; WM, white matter.

**Table 4:** Comparison of the inter-method reliability of volumetric measurements across the three volumetry software packages.

	Lt. Hemisphere			Rt. Hemisphere		
	NQ vs. FS	NQ vs. HAD	FS vs. HAD	NQ vs. FS	NQ vs. HAD	FS vs. HAD
**Cortical GM**						
Total	0.945 (0.914–0.965)	0.945 (0.914–0.965)	0.945 (0.913–0.965)	0.947 (0.916–0.966)	0.944 (0.912–0.964)	0.951 (0.923–0.969)
Non-AD	0.939 (0.888–0.966)	0.936 (0.884–0.965)	0.954 (0.917–0.975)	0.934 (0.880–0.964)	0.932 (0.876–0.962)	0.950 (0.910–0.973)
AD	0.970 (0.939–0.985)	0.876 (0.784–0.939)	0.897 (0.791–0.949)	0.990 (0.980–0.995)	0.954 (0.909–0.977)	0.974 (0.948–0.987)
**Cerebral WM**						
Total	1.000 (1.000–1.000)	0.983 (0.973–0.989)	0.983 (0.973–0.989)	1.000 (1.000–1.000)	0.978 (0.965–0.986)	0.978 (0.965–0.986)
Non-AD	1.000 (1.000–1.000)	0.990 (0.982–0.995)	0.990 (0.982–0.995)	1.000 (1.000–1.000)	0.988 (0.978–0.993)	0.988 (0.978–0.993)
AD	1.000 (1.000–1.000)	0.972 (0.944–0.986)	0.972 (0.944–0.986)	0.996 (0.992–0.998)	0.988 (0.976–0.994)	0.986 (0.972–0.993)
**Hippocampus**						
Total	0.918 (0.871–0.948)	0.724 (0.567–0.824)	0.842 (0.753–0.899)	0.917 (0.869–0.947)	0.621 (0.406–0.759)	0.807 (0.698–0.877)
Non-AD	0.904 (0.826–0.947)	0.600 (0.271–0.780)	0.780 (0.599–0.879)	0.886 (0.793–0.938)	0.523 (0.132–0.738)	0.784 (0.607–0.881)
AD	0.914 (0.826–0.958)	0.895 (0.787–0.948)	0.892 (0.782–0.947)	0.947 (0.892–0.974)	0.835 (0.665–0.918)	0.863 (0.723–0.932)
**Amygdala**						
Total	0.916 (0.869–0.947)	0.897 (0.838–0.934)	0.924 (0.881–0.952)	0.892 (0.830–0.931)	0.898 (0.840–0.935)	0.936 (0.900–0.959)
Non-AD	0.897 (0.812–0.943)	0.888 (0.795–0.938)	0.890 (0.800–0.940)	0.844 (0.715–0.914)	0.870 (0.762–0.928)	0.923 (0.860–0.958)
AD	0.909 (0.815–0.955)	0.887 (0.772–0.944)	0.967 (0.933–0.984)	1.000 (1.000–1.000)	0.998 (0.996–0.999)	0.999 (0.998–0.999)
**Caudate**						
Total	0.876 (0.805–0.821)	0.843 (0.753–0.900)	0.899 (0.842–0.936)	0.631 (0.420–0.764)	0.850 (0.764–0.904)	0.787 (0.666–0.864)
Non-AD	0.856 (0.783–0.921)	0.780 (0.600–0.879)	0.883 (0.787–0.936)	0.780 (0.600–0.879)	0.835 (0.700–0.909)	0.870 (0.764–0.929)
AD	0.903 (0.804–0.952)	0.880 (0.757–0.941)	0.915 (0.828–0.958)	0.999 (0.998–1.000)	0.997 (0.995–0.999)	0.998 (0.997–0.999)
**Putamen**						
Total	0.824 (0.724–0.888)	0.840 (0.749–0.898)	0.912 (0.862–0.944)	0.853 (0.770–0.906)	0.796 (0.680–0.870)	0.947 (0.916–0.966)
Non-AD	0.810 (0.655–0.896)	0.821 (0.675–0.902)	0.973 (0.851–0.985)	0.760 (0.563–0.868)	0.977 (0.958–0.987)	0.801 (0.639–0.891)
AD	0.728 (0.449–0.866)	0.869 (0.734–0.935)	0.762 (0.518–0.882)	1.000 (0.999–1.000)	0.998 (0.997–0.999)	0.999 (0.997–0.999)
**Pallidum**						
Total	0.117 (−0.385–0.437)	0.558(0.307–0.718)	0.778 (0.651–0.858)	0.077 (−0.447–0.412)	0.370 (0.012–0.598)	0.710 (0.546–0.815)
Non-AD	0.036 (−0.754–0.470)	0.613 (0.296–0.788)	0.792 (0.622–0.886)	0.365 (−0.156–0.651)	0.381 (−0.126–0.660)	0.916 (0.847–0.954)
AD	0.419 (−0.177–0.713)	0.446 (−0.122–0.726)	0.446 (−0.122–0.726)	−0.045 (−1.116–0.484)	0.573 (0.136–0.789)	0.232 (−0.554–0.621)
**Thalamus**						
Total	0.954 (0.923–0.971)	0.925 (0.882–0.952)	0.974 (0.960–0.984)	0.908 (0.855–0.941)	0.899 (0.842–0.936)	0.982 (0.972–0.989)
Non-AD	0.956 (0.920–0.976)	0.938 (0.886–0.966)	0.978 (0.960–0.988)	0.899 (0.815–0.944)	0.907 (0.832–0.949)	0.984 (0.971–0.991)
AD	0.907 (0.812–0.954)	0.791 (0.577–0.897)	0.928 (0.854–0.964)	1.000 (0.999–1.000)	0.998 (0.996–0.999)	0.999 (0.997–0.999)
**Cerebellum**						
Total	0.985 (0.977–0.990)	0.944 (0.912–0.964)	0.941 (0.908–0.963)	0.978 (0.965–0.986)	0.953 (0.927–0.970)	0.964 (0.943–0.977)
Non-AD	0.975 (0.955–0.986)	0.909 (0.834–0.950)	0.909 (0.834–0.950)	0.969 (0.944–0.983)	0.936 (0.883–0.965)	0.934 (0.880–0.964)
AD	0.988 (0.975–0.994)	0.947 (0.892–0.974)	0.942 (0.883–0.971)	0.979 (0.957–0.990)	0.954 (0.907–0.977)	0.976 (0.952–0.988)

Note—Data are presented as intraclass correlation coefficient (95% confidence interval); AD, Alzheimer’s disease; FS, FreeSurfer; GM, gray matter; HAD, Heuron AD; NQ, NeuroQuant; WM, white matter.

**Table 5:** Comparison of the effect size across the three volumetry software packages.

	Lt. Hemisphere			Rt. Hemisphere		
	NQ vs. FS	NQ vs. HAD	FS vs. HAD	NQ vs. FS	NQ vs. HAD	FS vs. HAD
**Cortical GM**						
Total	0.52	1.29	0.97	0.57	1.32	0.93
Non-AD	0.71	1.32	0.79	0.71	1.33	0.80
AD	0.40	2.12	1.91	0.57	2.22	1.83
**Cerebral WM**						
Total	0.66	0.29	0.35	0.87	0.36	0.47
Non-AD	0.62	0.19	0.43	0.81	0.25	0.54
AD	0.78	0.49	0.26	1.07	0.59	0.41
**Hippocampus**						
Total	0.33	0.03	0.40	0.37	0.78	0.48
Non-AD	0.14	0.54	0.48	0.16	0.33	0.20
AD	0.90	1.87	1.09	0.79	1.74	0.93
**Amygdala**						
Total	0.44	0.08	0.49	0.36	0.78	0.50
Non-AD	0.42	0.02	0.42	0.36	0.97	0.79
AD	0.59	0.21	0.72	0.63	0.86	0.32
**Caudate**						
Total	0.16	0.39	0.28	0.12	0.34	0.60
Non-AD	0.30	0.17	0.46	0.02	0.37	0.49
AD	0.05	0.34	0.47	0.29	0.33	0.75
**Putamen**						
Total	1.82	2.12	0.25	1.50	1.81	0.32
Non-AD	1.80	2.25	0.40	2.17	0.45	1.61
AD	2.31	2.21	<0.01	1.83	1.95	0.13
**Pallidum**						
Total	5.65	5.49	0.13	6.07	5.63	0.10
Non-AD	5.20	5.28	0.06	6.52	5.82	0.26
AD	6.93	6.17	0.43	2.25	2.01	0.46
**Thalamus**						
Total	0.73	1.06	0.34	0.10	1.15	0.06
Non-AD	0.98	1.04	0.02	1.11	1.13	0.02
AD	1.48	1.53	0.02	2	1.94	0.18
**Cerebellum**						
Total	0.10	0.64	0.56	0.17	0.53	0.36
Non-AD	0.14	0.69	0.57	0.18	0.58	0.43
AD	0.09	0.76	0.71	0.20	0.57	0.37

Note—AD, Alzheimer’s disease; FS, FreeSurfer; GM, gray matter; HAD, Heuron AD; NQ, NeuroQuant; WM, white matter.

## Data Availability

The datasets generated during and/or analyzed during the current study are available from the corresponding author on reasonable request.

## Supplementary Materials

The following supporting information can be downloaded at: https://www.mdpi.com/article/10.3390/medicina60050727/s1, Table S1: Comparison of the mean bias and 95% limits of agreement (LOA) for total intracranial volume between the three volumetry software packages; Table S2: Comparison of the mean bias and 95% limits of agreement (LOA) for measured volumes in each brain region between the three volumetry software packages

## References

[1] Giorgio A., De Stefano N. (2013). Clinical use of brain volumetry. J. Magn. Reson. Imaging.

[2] Brewer J.B., Magda S., Airriess C., Smith M.E. (2009). Fully-Automated Quantification of Regional Brain Volumes for Improved Detection of Focal Atrophy in Alzheimer Disease. Am. J. Neuroradiol..

[3] Song H., Lee S.A., Jo S.W., Chang S.-K., Lim Y., Yoo Y.S., Kim J.H., Choi S.H., Sohn C.-H. (2022). Agreement and Reliability between Clinically Available Software Programs in Measuring Volumes and Normative Percentiles of Segmented Brain Regions. Korean J. Radiol..

[4] Reid M.W., Hannemann N.P., York G.E., Ritter J.L., Kini J.A., Lewis J.D., Sherman P.M., Velez C.S., Drennon A.M., Bolzenius J.D. (2017). Comparing two processing pipelines to measure subcortical and cortical volumes in patients with and without mild traumatic brain injury. J. Neuroimaging.

[5] Ochs A.L., Ross D.E., Zannoni M.D., Abildskov T.J., Bigler E.D., Alzheimer’s Disease Neuroimaging Initiative (2015). Comparison of automated brain volume measures obtained with NeuroQuant® and FreeSurfer. J. Neuroimaging.

[6] Yim Y., Lee J.Y., Oh S.W., Chung M.S., Park J.E., Moon Y., Jeon H.J., Moon W.-J. (2021). Comparison of automated brain volume measures by NeuroQuant vs. Freesurfer in patients with mild cognitive impairment: Effect of slice thickness. Yonsei Med. J..

[7] Heo Y.J., Baek H.J., Skare S., Lee H.-J., Kim D.-H., Kim J., Yoon S. (2022). Automated brain volumetry in patients with memory impairment: Comparison of conventional and ultrafast 3d t1-weighted MRI sequences using two software packages. Am. J. Roentgenol..

[8] Ross D.E., Seabaugh J., Seabaugh J.M., Barcelona J., Seabaugh D., Wright K., Norwind L., King Z., Graham T.J., Baker J. (2022). Updated review of the evidence supporting the medical and legal use of NeuroQuant^®^ and NeuroGage^®^ in patients with traumatic brain injury. Front. Hum. Neurosci..

[9] Fischl B. (2012). FreeSurfer. Neuroimage.

[10] Sherfey J.S., Soplata A.E., Ardid S., Roberts E.A., Stanley D.A., Pittman-Polletta B.R., Kopell N.J. (2018). DynaSim: A MATLAB toolbox for neural modeling and simulation. Front. Neuroinform..

[11] Scarpazza C., Ha M., Baecker L., Garcia-Dias R., Pinaya W., Vieira S., Mechelli A. (2020). Translating research findings into clinical practice: A systematic and critical review of neuroimaging-based clinical tools for brain disorders. Transl. Psychiatry.

[12] Lee J.Y., Oh S.W., Chung M.S., Park J.E., Moon Y., Jeon H.J., Moon W.-J. (2021). Clinically available software for automatic brain volumetry: Comparisons of volume measurements and validation of intermethod reliability. Korean J. Radiol..

[13] Lee J.-Y., Park J.-E., Chung M.-S., Oh S.-W., Moon W.-J. (2021). Expert opinions and recommendations for the clinical use of quantitative analysis software for MRI-based brain volumetry. J. Korean Radiol. Soc..

[14] Shin D.H., Heo H., Song S., Shin N.Y., Nam Y., Yoo S.W., Kim J.S., Yoon J.H., Lee S.H., Sung Y.H. (2021). Automated assessment of the substantia nigra on susceptibility map-weighted imaging using deep convolutional neural networks for diagnosis of Idiopathic Parkinson’s disease. Park. Relat. Disord..

[15] McKhann G.M., Knopman D.S., Chertkow H., Hyman B.T., Jack C.R., Kawas C.H., Klunk W.E., Koroshetz W.J., Manly J.J., Mayeux R. (2011). The diagnosis of dementia due to Alzheimer’s disease: Recommendations from the National Institute on Aging-Alzheimer’s Association workgroups on diagnostic guidelines for Alzheimer’s disease. Alzheimer’s Dement..

[16] Ronald C.P., Glenn E.S., Stephen C.W., Robert J.I., Eric G.T., Emre K. (1999). Mild cognitive impairment. Arch. Neurol..

[17] Renvall V., Witzel T., Wald L.L., Polimeni J.R. (2016). Automatic cortical surface reconstruction of high-resolution T1 echo planar imaging data. Neuroimage.

[18] Landis J.R., Koch G.G. (1977). The measurement of observer agreement for categorical data. Biometrics.

[19] Olejnik S., Algina J. (2000). Measures of effect size for comparative studies: Applications, interpretations, and limitations. Contemp. Educ. Psychol..

[20] Cohen J. (1992). Quantitative Methods in Psychology: A Power Primer. Psychol. Bull..

[21] Ross D.E., Ochs A.L., Tate D.F., Tokac U., Seabaugh J., Abildskov T.J., Bigler E.D. (2018). High correlations between MRI brain volume measurements based on NeuroQuant® and FreeSurfer. Psychiatry Res. Neuroimaging.

[22] Klauschen F., Goldman A., Barra V., Meyer-Lindenberg A., Lundervold A. (2009). Evaluation of Automated Brain MR Image Segmentation and Volumetry Methods.

[23] Chung J., Kim H., Moon Y., Moon W.-J. (2020). Comparison of vendor-provided volumetry software and NeuroQuant using 3D T1-weighted images in subjects with cognitive impairment: How large is the inter-method discrepancy?. Investig. Magn. Reson. Imaging.

[24] Nestor S.M., Gibson E., Gao F.-Q., Kiss A., Black S.E., Alzheimer’s Disease Neuroimaging Initiative (2013). A direct morphometric comparison of five labeling protocols for multi-atlas driven automatic segmentation of the hippocampus in Alzheimer’s disease. Neuroimage.

[25] Fischl B., Salat D.H., Busa E., Albert M., Dieterich M., Haselgrove C., van der Kouwe A., Killiany R., Kennedy D., Klaveness S. (2002). Whole brain segmentation: Automated labeling of neuroanatomical structures in the human brain. Neuron.

[26] Kanda T., Nakai Y., Aoki S., Oba H., Toyoda K., Kitajima K., Furui S. (2016). Contribution of metals to brain MR signal intensity: Review articles. Jpn. J. Radiol..

[27] Lee J., Lee J.Y., Oh S.W., Chung M.S., Park J.E., Moon Y. (2021). Evaluation of reproducibility of brain volumetry between commercial software, inbrain and established research purpose method, FreeSurfer. J. Clin. Neurol..

[28] Jack C.R. (2012). Continuing Medical Education: Alzheimer Disease: New Concepts on Its Neurobiology and the Clinical Role Imaging Will Play. Radiology.

[29] Tae W.S., Kim S.S., Lee K.U., Nam E.-C., Kim K.W. (2008). Validation of hippocampal volumes measured using a manual method and two automated methods (FreeSurfer and IBASPM) in chronic major depressive disorder. Neuroradiology.

[30] Morey R.A., Petty C.M., Xu Y., Hayes J.P., Wagner H.R., Lewis D.V., LaBar K.S., Styner M., McCarthy G. (2009). A comparison of automated segmentation and manual tracing for quantifying hippocampal and amygdala volumes. Neuroimage.

[31] Guenette J.P., Stern R.A., Tripodis Y., Chua A.S., Schultz V., Sydnor V.J., Somes N., Karmacharya S., Lepage C., Wrobel P. (2018). Automated versus manual segmentation of brain region volumes in former football players. Neuroimage Clin..

