# Supplementary material for: Comparison of Inter-Method Agreement and Reliability for Automatic Brain Volumetry Using Three Different Clinically Available Software Packages
Source: Medicina (Kaunas). 2024 Apr 27;60(5):727. doi: 10.3390/medicina60050727 (PMC11122718; doi:10.3390/medicina60050727)
Supplement: Supplementary file 1 [file medicina-60-00727-s001.zip › medicina-2963092-supplementary.pdf]

Supportive/Supplementary Material

Table S1. Comparison of the mean bias and 95% limits of agreement (LOA) for total intracranial volume between the three volumetry software packages

	NQ vs. FS		NQ vs. HAD		FS vs. HAD	
	Mean Bias	95% LOA	Mean Bias	95% LOA	Mean Bias	95% LOA
Total	12.96	-90.72, 116.65	45.90	-104.97, 196.78	32.95	-74.41, 140.30
Non-AD	27.20	-82.55, 136.95	53.67	-127.12, 234.46	26.47	-108.71, 161.65
AD	-6.55	-88.42, 75.51	35.32	-59.88, 130.53	41.78	-3.73, 87.28

Note— AD, Alzheimer's disease; FS, FreeSurfer; HAD, Heuron AD; LOA, limits of agreement; NQ, NeuroQuant

Table S2. Comparison of the mean bias and 95% limits of agreement (LOA) for measured volumes in each brain region between the three volumetry software packages.

	Lt. hemisphere						Rt. hemisphere					
	NQ vs. FS		NQ vs. HAD		FS vs. HAD		NQ vs. FS		NQ vs. HAD		FS vs. HAD	
	**Mean Bias**	**95% LOA**	**Mean Bias**	**95% LOA**	**Mean Bias**	**95% LOA**	**Mean Bias**	**95% LOA**	**Mean Bias**	**95% LOA**	**Mean Bias**	**95% LOA**
**Cortical GM**												
Total	15.87	-11.28, 43.03	41.96	13.38, 70.54	26.09	1.81, 50.36	17.30	-9.56, 44.17	42.21	13.25, 71.18	24.90	2.14, 47.66
Non-AD	21.78	-7.19, 50.76	42.69	11.66, 73.71	20.91	-0.68, 42.49	21.78	-8.17, 51.72	42.09	10.70, 73.49	20.32	-1.40, 42.03
AD	7.82	-5.41, 21.05	40.98	15.77, 66.18	33.16	12.32, 53.99	11.22	-4.56, 27.00	42.37	16.62, 68.12	31.15	13.00, 49.31
**Cerebral WM**												
Total	17.00	16.99, 17.01	7.72	-5.79, 21.23	-9.28	-22.79, 4.23	22.40	22.40, 22.41	9.66	-6.00, 25.32	-12.74	-28.40, 2.92
Non-AD	17.00	16.99, 17.00	5.28	-5.24, 15.80	-11.72	-22.24, -1.20	22.40	22.40, 22.41	7.08	-4.98, 19.14	-15.33	-27.39, -3.27
AD	17.00	17.00, 17.01	11.05	-3.48, 25.57	-5.95	-20.48, 8.57	22.40	22.40, 22.41	13.18	-4.28, 30.64	-9.22	-26.68, 8.24
**Hippocampus**												
Total	-0.23	-0.99, 0.53	-0.57	-1.74, 0.61	-0.34	-1.08, 0.42	-0.28	-1.14, 0.58	-0.56	-2.03, 0.92	-0.28	-1.16, 0.61
Non-AD	-0.10	-0.92, 0.72	-0.34	-1.65, 0.97	-0.24	-1.08, 0.60	-0.12	-1.03, 0.79	-0.22	-1.72, 1.27	-0.10	-0.92, 0.72

AD	-0.41	-0.91, 0.95	-0.88	-1.45, -0.31	-0.47	-0.99, 0.06	-0.50	-1.06, 0.06	-1.01	-1.88, -0.15	-0.51	-1.27, 0.25
**Amygdala**												
Total	0.14	-0.21, 0.50	-0.03	-0.48, 0.42	-0.17	-0.54, 0.19	-0.11	-0.45, 0.23	-0.26	-0.64, 0.13	-0.15	-0.43, 0.14
Non-AD	0.14	-0.26, 0.54	-0.01	-0.52, 0.50	-0.15	-0.59, 0.29	-0.11	-0.50, 0.29	-2.30	-0.71, 0.11	-0.19	-0.46, 0.07
AD	0.15	-0.15, 0.44	-0.06	-0.41, 0.28	-0.21	-0.41, 0.00	-0.11	-0.35, 0.13	-0.20	-0.51, 0.11	-0.09	-0.35, 0.18
**Caudate**												
Total	-0.98	-0.92, 0.72	-0.24	-1.13, 0.66	-0.14	-0.73, 0.45	0.07	-1.13, 1.28	-0.21	-1.08, 0.66	-0.28	-1.04, 0.48
Non-AD	-0.19	-1.06, 0.68	-0.27	-1.26, 0.72	-0.08	-0.68, 0.53	-0.002	-0.97, 0.96	-0.22	-1.10, 0.67	-0.21	-0.79, 0.36
AD	0.03	-0.66, 0.71	-0.20	-0.95, 0.56	-0.23	-0.75, 0.30	0.17	-1.29, 1.64	-0.20	-1.05, 0.66	-0.37	-1.32, 0.57
**Putamen**												
Total	1.45	0.24, 0.27	1.62	0.51, 2.73	0.16	-0.60, 0.93	1.23	0.08, 2.38	1.45	0.16, 2.74	0.22	-0.40, 0.83
Non-AD	1.48	0.18, 2.77	1.76	0.57, 2.96	0.29	-0.16, 0.73	1.29	-0.04, 2.62	1.62	0.20, 3.03	0.33	-0.10, 0.76
AD	1.42	0.31, 2.54	1.42	0.56, 2.28	-0.003	-0.97, 0.96	1.16	0.31, 2.00	1.22	0.28, 2.16	0.07	-0.64, 0.77
**Pallidum**												
Total	-1.43	-2.10, -0.76	-1.39	-1.93, -0.85	0.04	-0.48, 0.57	-1.42	-2.05, -0.78	-1.39	-1.99, -0.79	0.03	-0.55, 0.60
Non-AD	-1.35	-2.06, -0.64	-1.37	-1.91, -0.83	-0.02	-0.54, 0.50	-1.33	-1.82, -0.84	-1.40	-1.98, -0.82	-0.07	-0.36, 0.23

AD	-1.55	-2.08, -1.02	-1.42	-1.97, -0.88	0.13	-0.37, 0.62	-1.53	-2.27, -0.79	-1.37	-2.00, -0.74	0.16	-0.59, 0.91
**Thalamus**												
Total	1.05	0.20, 1.90	1.06	0.03, 2.09	0.01	-0.55, 0.57	1.12	0.02, 2.23	1.07	-0.04, 2.18	-0.05	-0.05, 0.40
Non-AD	1.12	0.21, 2.02	1.14	0.10, 2.17	0.02	-0.56, 0.59	1.15	-0.08, 2.39	1.13	-0.01, 2.27	-0.02	-0.49, 0.44
AD	0.96	0.22, 1.70	0.95	-0.06, 1.97	-0.01	-0.56, 0.54	1.08	0.17, 1.99	0.99	-0.06, 2.04	-0.09	-0.54, 0.36
**Cerebellum**												
Total	0.74	-2.67, 4.15	4.89	-2.07, 11.85	4.15	-2.70, 11.01	1.32	-3.04, 5.69	4.09	-2.29, 10.48	2.77	-2.81, 8.34
Non-AD	0.87	-2.90, 4.63	4.37	-2.82. 11.57	3.51	-3.41, 10.43	1.18	-3.31, 5.68	3.90	-2.59, 10.39	2.72	-3.50, 8.94
AD	0.56	-2.32, 3.44	5.60	-0.88, 12.08	5.03	-1.45, 11.52	1.51	-2.70, 5.73	4.35	-1.95, 10.66	2.84	-1.80, 7.48

Note— AD, Alzheimer's disease; FS, FreeSurfer; GM, gray matter; HAD, Heuron AD; LOA, limits of agreement; NQ, NeuroQuant; WM, white matter
